# Development and Evaluation of the Joseph-RATA (Rubrics Ask-Tell-Ask) Structured Feedback Model in Undergraduate Pharmacology

**DOI:** 10.7759/cureus.114897

**Published:** 2026-08-20

**Authors:** Girish Joseph, Neena Bhatti, Dinesh K Badyal, Aroma Oberoi, Ajay Kumar

**Affiliations:** 1 Pharmacology and Therapeutics, Christian Medical College & Hospital, Ludhiana, IND; 2 Microbiology, Christian Medical College & Hospital, Ludhiana, IND; 3 Biochemistry, Christian Medical College & Hospital, Ludhiana, IND

**Keywords:** ask-tell-ask, assessment, competency-based medical education, curriculum, formative feedback, joseph-rata, medical education, pharmacology, rubrics

## Abstract

Background: Effective feedback is central to competency-based medical education (CBME), yet structured formats for delivering formative feedback remain uncommon in many undergraduate settings. At our institution, students reported dissatisfaction with existing feedback practices, which were largely limited to the communication of marks without actionable guidance. A transparent, standardized, and learner-centered feedback framework aligned with CBME principles was therefore required.

Objective: The objective of this study is to develop, validate, and implement a structured feedback method for formative assessment in undergraduate pharmacology and to evaluate the perceptions of students and faculty regarding its use.

Methods: This prospective interventional study was conducted over six months in the Department of Pharmacology at Christian Medical College and Hospital, Ludhiana, India. All students of the 2023 and 2024 MBBS batches (n = 200) were included. A structured Rubric-Ask-Tell-Ask (RATA) feedback model was developed by a departmental core committee, validated by Medical Education Unit members and by students of the 2022 MBBS batch, and implemented across six formative assessments. Perceptions were assessed using a 14-item Likert-type questionnaire, a single binary recommendation item, focus group discussions with students, and semi-structured interviews with faculty. Quantitative data were summarized using descriptive statistics, while qualitative data were analyzed thematically.

Results: Dissatisfaction with the previous feedback practice was reported by 168 of 200 students (84.0%), with a mean item score of 2.02 ± 0.69 out of 5. After implementation of the structured model, students reported consistently positive perceptions across all evaluated domains following implementation of the structured feedback model. Continuation of the model was recommended by 193 of 200 students (96.5%). All five participating faculty members endorsed continuation, with a mean faculty rating of 4.69 out of 5. Qualitative themes included improved clarity of performance expectations, transparency in marking, facilitation of self-directed learning, and acceptability of the model within large-group teaching.

Conclusions: The Joseph-RATA structured feedback model demonstrated high acceptability, feasibility, and perceived educational value among both students and faculty, supporting its integration into competency-based undergraduate curricula.

## Introduction

Feedback is a fundamental component of education and learning. It has been defined as specific information about the comparison between a trainee's observed performance and a standard, given with the intent to improve the trainee's performance [1]. Feedback is essential to medical education because it fosters lifelong learning, offers behavioral insight, enhances knowledge and skills, and motivates learners to improve. When teachers do not provide feedback, students must rely on their own appraisals to identify areas of strength and areas requiring growth [2].

The concept of feedback is central to formative assessment, which occurs during the learning process. This stands in contrast to summative assessment, which typically occurs at the conclusion of a learning episode and seeks to judge the student's level of skill or knowledge. Feedback based solely on summative assessment is therefore less productive, since it is delivered after the opportunity for improvement has passed [3]. In the medical context, feedback supports the development of accurate self-assessment, and a deficiency of feedback may extend beyond individual self-improvement to eventually influence patient care. Learners who do not receive sufficient feedback on their performance may lose the opportunity to grow and to reflect [4].

Only effective feedback has genuine impact and acceptability. Effective feedback should be timely and delivered as close to the event as possible for maximum impact; specific, with concrete examples; provided by first-hand observers rather than through the interpretations of others; collaborative between facilitator and learner; and actionable, identifying the changes required for future improvement [5].

Several methods of delivering feedback are in common use, including the sandwich method, the Ask-Tell-Ask (ATA) method, and Pendleton's method [6]. In the sandwich method, two positive statements surround a middle segment that may be perceived as negative [5]. The impact of sandwich feedback is diminished because the learner anticipates the coming criticism and consequently discounts the constructive content [7]. Furthermore, the surrounding positive comments carry little constructive value and may be perceived as patronizing [5].

The ATA method has three parts. The facilitator first asks the learner to self-assess their performance, then provides specific feedback, and finally asks the learner about their understanding of the feedback and their action plan for improvement [6,8]. This bidirectional approach promotes learner engagement and reflection and has been used in undergraduate health-professions education [8]. However, ATA primarily provides a conversational structure for delivering feedback and does not inherently prescribe standardized performance descriptors or explicit criteria against which learner performance can be judged. Consequently, the specificity and consistency of feedback may depend on how individual assessors interpret and communicate performance. Incorporating a rubric alongside ATA can address this gap by providing predefined performance descriptors and transparent criteria, thereby establishing a common reference point for learner self-reflection and facilitator feedback. Recent work on standardized feedback evaluation has similarly highlighted the value of rubric-based frameworks for promoting consistency and structured assessment of verbal feedback [9]. Thus, the Joseph-RATA model combines the interactive and reflective strengths of ATA with the standardization and transparency provided by rubric-based assessment.

Pendleton's method is a modification of the sandwich model in which the facilitator's comments are preceded by the learner's account of what was done well and what could be improved. It is a comparatively inflexible method, and its effect is offset by the rigidity of the conversation and by the learner's expectation of criticism [10]. Although educators recognize the necessity of feedback, it remains underused, in part because there is no structured format to guide delivery; consequently, feedback is either omitted or delivered ineffectively [2].

In order to make medical education more learner-centric, patient-centric, and internationally relevant, the National Medical Commission (NMC), through the Competency-Based Medical Education (CBME) Guidelines 2024, has placed strong emphasis on feedback, and the current curriculum places greater weight on formative assessment, for which feedback is essential [11]. A recognized deficiency in the current MBBS curriculum is the absence of a framework for providing feedback to students. Facilitators are also seldom trained in how to deliver feedback, and no standardized structure exists. The present project was designed to create a structured feedback format that would enable facilitators to provide timely feedback, thereby improving student performance, engagement with the subject, and skill development.

The aim of this study was to develop and implement a structured feedback method for formative assessment in undergraduate pharmacology. The specific objectives were: (1) to sensitize faculty regarding the need for timely feedback; (2) to train faculty to use the validated structured feedback tool; (3) to implement the validated structured feedback; and (4) to evaluate the perceptions of faculty and students regarding the structured feedback.

## Materials and methods

Study design and setting

This prospective interventional project was conducted in the Department of Pharmacology, Christian Medical College and Hospital, Ludhiana, Punjab, India, as part of a six-month FAIMER (Foundation for Advancement of International Medical Education and Research) project. The Joseph-RATA structured feedback intervention was planned as a longitudinal curricular strategy, with six formative assessment sessions scheduled between the second and ninth months of the professional year.

Participants and sampling

The study population comprised all students enrolled in the 2023 and 2024 MBBS batches (n = 200), as the Joseph-RATA structured feedback intervention was incorporated into the routine undergraduate pharmacology curriculum and was applicable to the entire cohort. Universal sampling (complete enumeration) was therefore employed. All students enrolled in these batches who were exposed to the intervention during the study period were eligible for inclusion; students from other batches or those not exposed to the intervention during the study period were outside the study population. The faculty evaluation included all five faculty members who were directly involved in implementing and delivering the Joseph-RATA intervention during the study period; therefore, no separate faculty sampling procedure was undertaken. All five participants were faculty members.

Development and validation of the intervention

A core committee was constituted within the Department of Pharmacology to design and implement a structured formative feedback model suitable for undergraduate CBME. The committee conducted structured brainstorming sessions for faculty sensitization and for the development of the structured feedback tool after reviewing the literature on formative feedback, CBME, structured rubrics, and established feedback models such as Ask-Tell-Ask. The Joseph-RATA (Rubrics-Ask-Tell-Ask) framework was subsequently developed by integrating rubric-based assessment with reflective faculty-student dialogue to promote standardized, transparent, and learner-centered feedback.

**Figure 1:**
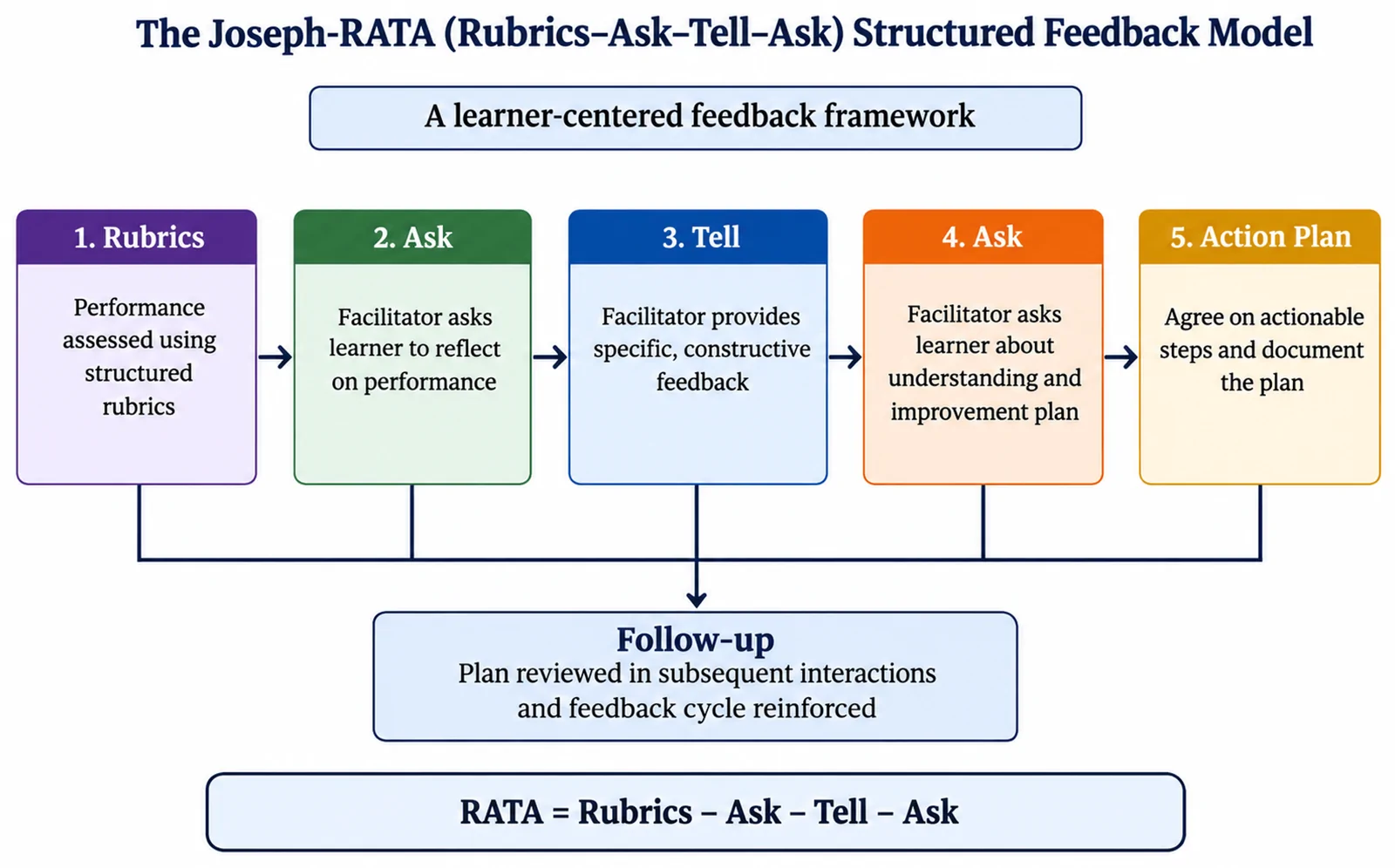
Joseph-RATA (Rubrics Ask-Tell-Ask) Source: MS Paint and MS PowerPoint

The draft structured feedback tool and perception questionnaires were independently reviewed by five members of the institutional Medical Education Unit (MEU) for clarity, relevance, comprehensiveness, feasibility, and alignment with competency-based learning objectives. Suggestions from the experts were incorporated through iterative revisions. The revised instruments were subsequently reviewed and reviewed for comprehensibility by five students from the 2022 MBBS batch, who were not included in the final study, to assess comprehensibility, feasibility, and ease of administration. Minor modifications in wording and formatting were undertaken based on their feedback before final implementation. Following completion of the development and validation process, the Joseph-RATA structured feedback model was implemented for all eligible students of the 2023 and 2024 MBBS batches and used across six formative assessment sessions during the study period. A conceptual representation of the Joseph-RATA framework is shown in Figure 1.

The Joseph-RATA model and its delivery

The intervention was incorporated into six formative assessment sessions conducted at different stages of the academic year. Following the second-month theory assessment, feedback was provided using the rubric-based component in a large-group format. The third-month internal assessment was followed by individual ATA feedback, in which the learner first reflected on their performance, the faculty member provided specific feedback, and the learner subsequently identified an action plan for improvement. The fourth-month practical assessment was followed by rubric-based feedback delivered in a large-group format. The fifth-month theory assessment again used the rubric-based approach in a large-group setting. The seventh-month internal assessment constituted a second, separate application of the ATA method and was similarly followed by one-on-one feedback using the same self-assessment, faculty feedback, and learner reflection/action-plan sequence. Finally, the ninth-month practical assessment was followed by rubric-based feedback delivered in a large-group format. Thus, the two internal assessments represented separate assessment events at different time points, with the same ATA feedback sequence applied individually after each assessment, whereas rubric-based feedback was used for the theory and practical assessments delivered in the large-group format. The schedule of delivery, the corresponding assessment type, the feedback method used, and the group format are presented in Table 1.

**Table 1:** Timeline of structured feedback delivery across the professional year Months denote the time elapsed since the start of the intervention, with the month representing the duration of the commencement of the professional year

Timeline	Type of assessment	Feedback method	Group format
Second month	Theory assessment	Rubrics	Large group
Third month	Internal assessment	Ask-Tell-Ask	One-on-one
Fourth month	Practical assessment	Rubrics	Large group
Fifth month	Theory assessment	Rubrics	Large group
Seventh month	Internal assessment	Ask-Tell-Ask	One-on-one
Ninth month	Practical assessment	Rubrics	Large group

The six-month duration refers to the FAIMER project period during which the development, implementation, and evaluation activities for the project were undertaken. The Joseph-RATA intervention itself was planned as a longitudinal curricular strategy extending across the professional year, with six feedback sessions scheduled at the second, third, fourth, fifth, seventh, and ninth months. Thus, the six-month project duration and the nine-month curricular intervention timeline represent different time frames.

In the ATA component, the facilitator begins by asking the learner to share their own perspective on their performance; the facilitator then tells the learner specific feedback; finally, the facilitator asks the learner about their understanding of the feedback and their action plan for improvement. In the rubric component, each assessed element was graded according to the percentage of marks scored, using the descriptors shown in Appendix 1 and 2.

Evaluation

Evaluation was carried out for all stakeholders. Students completed a 14-item questionnaire using a five-point Likert scale (1 = strongly disagree to 5 = strongly agree), together with a single binary item on whether the method should be continued. Item 1 assessed satisfaction with the feedback practice that preceded the intervention, and items 2 to 14 assessed the domains of the newly introduced framework. Faculty completed a parallel questionnaire and additionally participated in semi-structured interviews. A focus group discussion (FGD) was conducted with students. The FGD guide was developed a priori, before analysis of the questionnaire responses. The discussion was designed as a complementary qualitative component to explore students' experiences with the structured feedback model, including perceived usefulness, clarity, feasibility, acceptability, and areas for improvement. The FGD questions were therefore not based on or modified according to the subsequent quantitative questionnaire findings. All five faculty members who implemented the Joseph-RATA intervention participated in semi-structured interviews after completion of the study. An interview guide was used to explore their perceptions regarding the feasibility, acceptability, educational value, implementation challenges, and potential refinements of the structured feedback model. Each interview lasted approximately 20-30 minutes, was audio-recorded with participant consent, transcribed verbatim, and analyzed thematically.

Qualitative data collection

Following completion of the intervention, one FGD comprising ten purposively selected students representing different levels of academic performance was conducted. Participants were selected to ensure diversity of experiences with the intervention. The discussion was facilitated by a faculty member not involved in routine assessment, using a semi-structured interview guide. The session lasted approximately 30 minutes and was audio-recorded with participant consent.

Statistical analysis

Quantitative data from the perception questionnaires were summarized using descriptive statistics. Results are presented as frequencies, percentages, means, and standard deviations, as appropriate. The distribution of responses was summarized to describe students' and faculty perceptions regarding the structured feedback model. Qualitative data obtained from the FGD and the faculty interviews were audio-recorded, transcribed verbatim, and analyzed using NVivo (QSR International, Melbourne, Australia; licensed version). A thematic analysis approach was employed. Two investigators independently reviewed and coded the transcripts. Differences in coding were resolved through discussion until consensus was reached. Codes were subsequently grouped into categories, from which overarching themes were generated using an inductive thematic analysis approach. Triangulation of quantitative and qualitative findings was performed to enhance interpretive validity.

Ethical considerations

The study was conducted after obtaining approval from the Institutional Ethics Committee (Biomedical and Health Research) of Christian Medical College and Hospital, Ludhiana (approval number CMC/402). A waiver of consent was sought for the educational intervention itself, since the intervention formed part of the routine MBBS curriculum and all students were exposed to the method; permission for implementation was obtained from the departmental and institutional authorities. Participation in the perception survey, FGD, and faculty interviews was voluntary, and written informed consent was obtained from all participants prior to data collection. Participants received a detailed participant information sheet explaining the purpose of the study, the procedures involved, confidentiality measures, and their right to withdraw at any stage without academic consequence. All responses were anonymized and coded to maintain confidentiality. Audio recordings and transcripts were stored securely with restricted access. The study adhered to the ethical principles of autonomy, confidentiality, and academic integrity. This manuscript was prepared with reference to the Standards for Quality Improvement Reporting Excellence (SQUIRE 2.0) guidelines, where applicable, to enhance the completeness and transparency of reporting.

## Results

Baseline perception of the previous feedback practice

A total of 200 students participated in the perception survey following implementation of the structured feedback strategy, yielding a response rate of 100%. Of the respondents, 112/200 (56.0%) were female and 88/200 (44.0%) were male. To determine whether a change in strategy was warranted, students were asked to rate the adequacy of the feedback practices that preceded the intervention. This item received the lowest score in the survey, with a mean of 2.02 ± 0.69. A substantial majority of participants (168 of 200, 84.0%) disagreed or strongly disagreed that the earlier approach met their learning needs, while only 9/200 (4.5%) expressed agreement. This finding demonstrated clear dissatisfaction with the traditional system and validated the need for a structured, transparent method of feedback delivery.

Perception of the structured feedback model

In contrast to the baseline item, responses to items 2 to 14, which evaluated the various domains of the newly introduced framework, were consistently high. The mean score across these items was 4.12 out of 5, indicating strong approval. Several domains were rated particularly highly: item 3 (mean 4.45), item 4 (mean 4.39), item 5 (mean 4.34), and item 14 (mean 4.34) reflected very positive perceptions of the clarity, usefulness, and organization of the feedback. The lowest rating among the intervention items was observed for item 13 (mean 3.56 ± 1.05), although the mean remained above the neutral midpoint of the five-point Likert scale. Item-wise mean scores are shown in Figure 1. Standard deviations for most items were below 0.8, suggesting limited variability in responses and indicating that the method was implemented uniformly across assessors.

**Figure 2:**
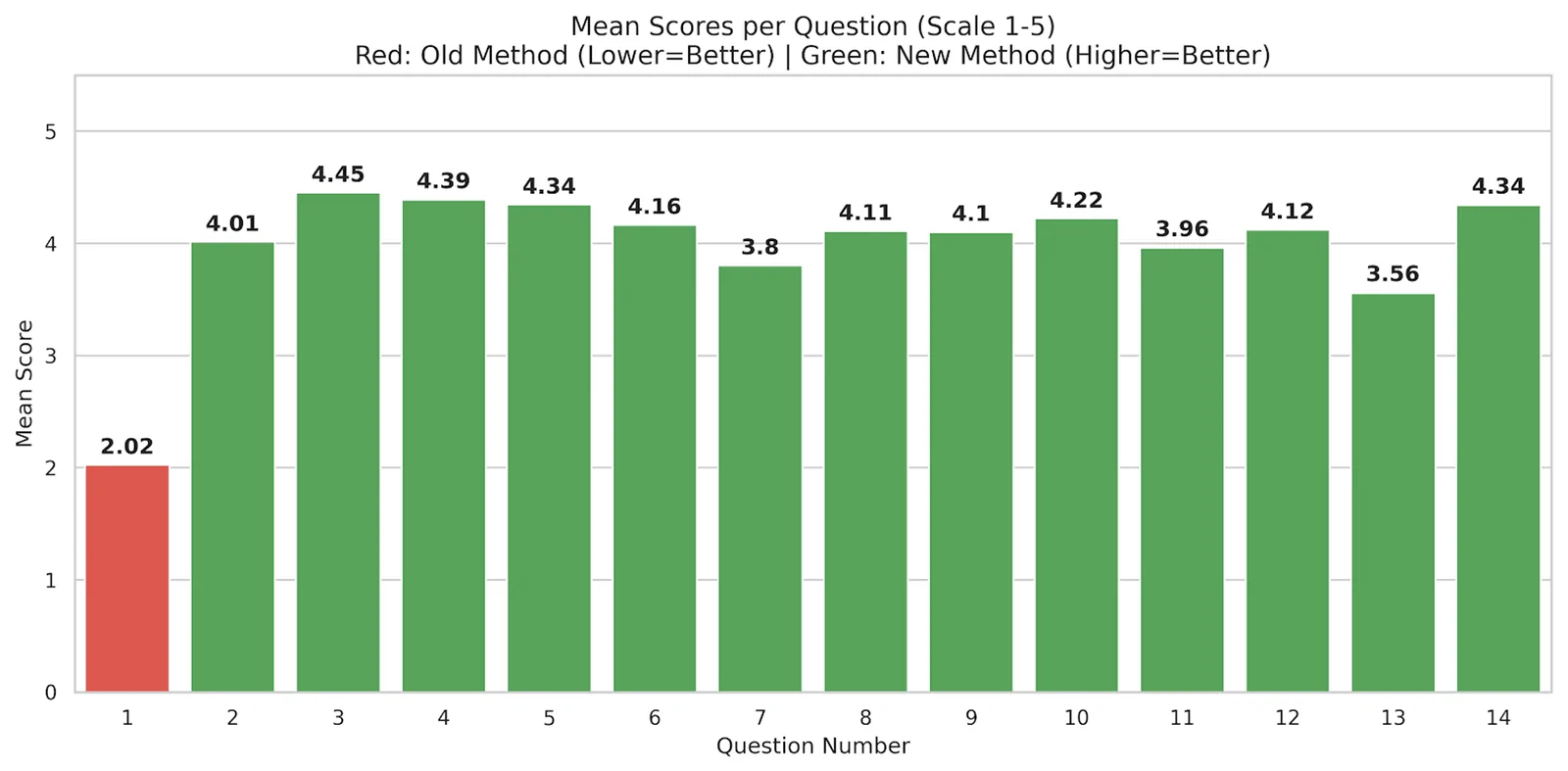
Item-wise mean perception scores for the structured feedback questionnaire (scale 1-5) Item 1 (red) assessed the previous unstructured feedback practice; items 2-14 (green) assessed the Joseph-RATA structured feedback model. RATA: Rubrics Ask-Tell-Ask

Distribution of responses

**Figure 3:**
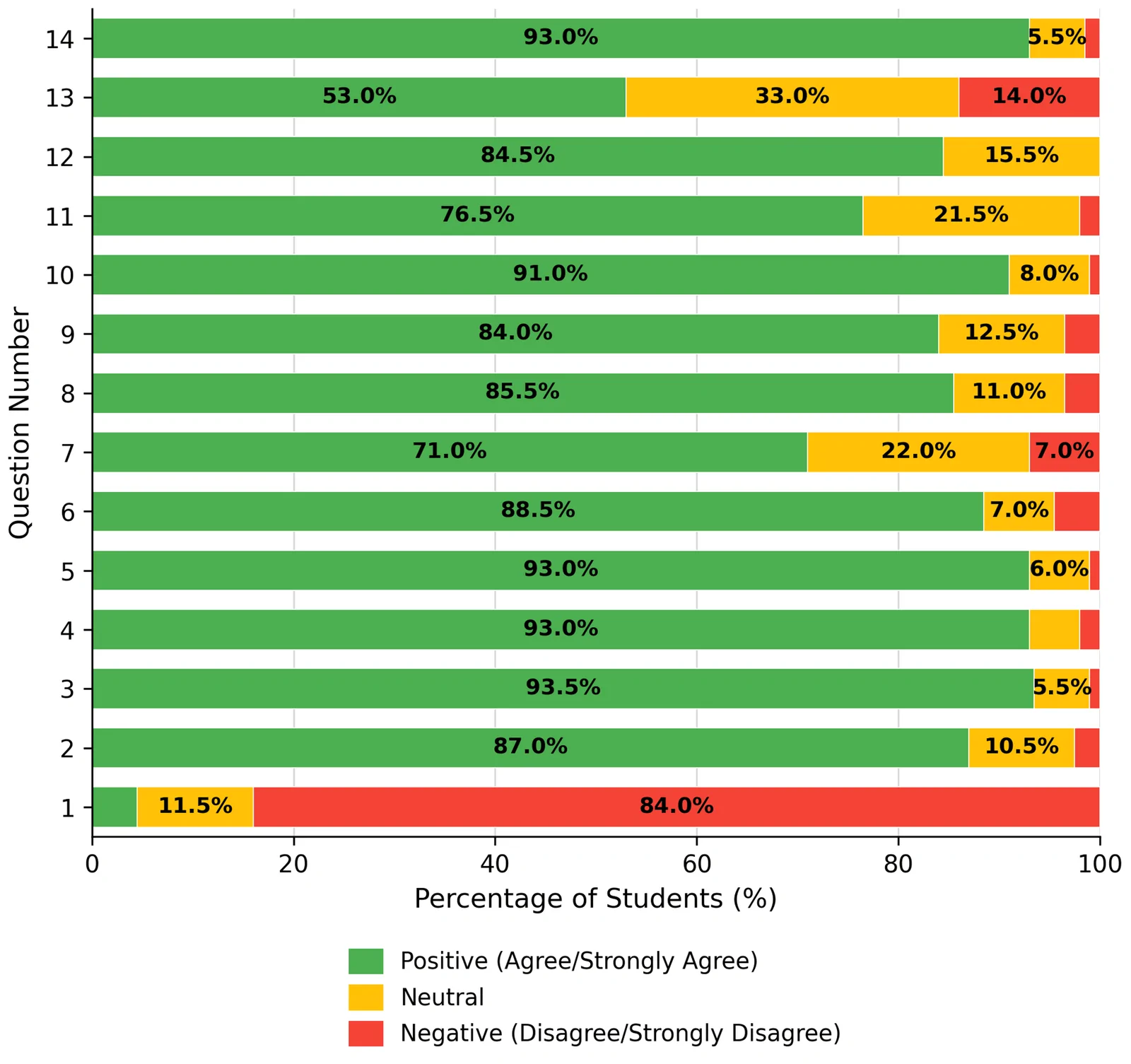
Percentage distribution of student responses for each questionnaire item Green: positive (agree or strongly agree); yellow: neutral; red: negative (disagree or strongly disagree)

Analysis of the response distribution further reinforced the strong acceptance of the intervention. For nearly all performance-related items, more than 150/200 (>75%) participants selected agree or strongly agree. In sharp contrast, 168/200 (84.0%) of students expressed dissatisfaction (disagree or strongly disagree) with the feedback provided before implementation of the structured method. The full percentage distribution of responses is shown in Figure 3.

Overall acceptability and recommendation

To evaluate the overall success of the implementation, students were asked to provide a binary recommendation (yes or no) on whether the validated structured feedback method should be continued (item 15). The results demonstrated near-universal acceptance: 193 of 200 participants (96.5%) voted 'yes', while 7/200 (3.5%) voted 'no'. This near-unanimous endorsement highlights strong learner acceptance and perceived value.

Qualitative evaluation through focus group discussion

To obtain deeper insight into learner experiences with the structured feedback intervention, an FGD was conducted with students who had undergone the method. The discussion explored perceptions regarding usefulness, clarity, feasibility, and areas for improvement. The conversation was audio-recorded, transcribed, and subjected to thematic analysis; codes were iteratively grouped into categories, which were subsequently synthesized into overarching themes. The analysis yielded themes relating to the perceived need for structured feedback, understanding of performance, promotion of self-directed learning, personalization despite large-group delivery, transparency in marking, applicability across subjects, and recommendations for refinement. Students also described the feedback as more personalized and less threatening, with reduced embarrassment and defensiveness despite the assessment context. A summary of the themes is presented in Table 2.

**Table 2:** Themes derived from thematic analysis of the student focus group discussion Source: Created by the authors after thematic analysis

Theme	Summary
Perceived need for structured feedback	Students reported that earlier feedback practices were largely limited to marks or isolated corrections, offering little direction for improvement. The new framework was seen as addressing a long-standing gap by organizing expectations and guiding performance enhancement.
Clarity in understanding performance	Participants emphasized that the method helped them understand how marks were awarded and what specific deficiencies existed. By linking score ranges with descriptors, the process converted evaluation into meaningful guidance.
Facilitation of self-directed learning	Learners felt empowered to independently identify strengths and weaknesses. High-scoring areas required minimal revision, while low-scoring domains could be selectively targeted, making preparation more efficient.
Personalization within large-group settings	Although feedback was delivered collectively, students perceived it as individualized. Because interpretation depended on one's own score, embarrassment and defensiveness were minimized, creating psychological safety.
Transparency and fairness in marking	The explicit criteria improved trust in faculty decisions. Students appreciated knowing the rationale behind awarded marks, which reduced ambiguity and perceptions of subjectivity.
Transferability across subjects	Participants believed that the framework could be adopted in other disciplines. The common assessment components across examinations made the model easy to generalize and repeatedly apply.
Desire for additional academic guidance	Some learners felt that while the structure was helpful, greater clarity regarding content expectations, sequencing of answers, and examiner priorities would further enhance usefulness.
Need for exam-strategy integration	Students suggested incorporating advice on time management and optimal answer length, noting that performance could be affected despite adequate knowledge if time was poorly distributed.
Importance of longitudinal implementation	Participants believed the method would have greater impact if used consistently after every assessment. Repetition would allow monitoring of trends and reinforce improvement strategies.

Faculty perceptions

The faculty survey (n = 5) revealed unanimous approval of the new method. The overall mean faculty rating was 4.69 out of 5, with 5/5 (100%) of responses falling into the agree or strongly agree categories across all parameters, indicating high confidence in the training received and in the utility of the tool. The lowest mean score recorded for any single parameter was 4.20 (items 2 and 5), which still fell within the agree category. All 5/5 respondents (100%) voted yes to recommend continued implementation of the validated structured feedback model.

To explore faculty perspectives on the Joseph-RATA structured feedback model, semi-structured interviews and a parallel survey were conducted with participating faculty members. The interviews examined perceptions regarding the need for structured feedback, feasibility of implementation, transparency in assessment, and overall educational impact. Transcripts were analyzed using thematic analysis, and recurring patterns were grouped into overarching themes, summarized in Table 3.

**Table 3:** Themes derived from thematic analysis of faculty interviews Source: Created by the authors after thematic analysis

Theme	Summary
Recognition of need	Traditional feedback lacked structure; the model addressed existing gaps.
Transparency and standardization	Explicit criteria improved fairness and reduced variability.
Reflective learning	The model encouraged self-assessment and focused preparation.
Feasibility	The approach was practical even in large batches.
Faculty accountability	Structured marking improved evaluator consistency.
Transferability	The model was considered applicable across disciplines with minor adaptation.
Implementation challenges	Time and training requirements were identified as constraints.
Suggested refinements	Incorporation of examination strategy and longitudinal use were suggested.

## Discussion

The present study demonstrates that implementation of the Joseph-RATA structured feedback model was associated with high learner acceptance, improved clarity of performance expectations, and enhanced perceived educational value. The findings support the growing body of literature emphasizing the critical role of structured, timely, and actionable feedback in CBME.

Our baseline findings demonstrated substantial dissatisfaction with previous feedback practices, with 84% of students reporting that the traditional approach did not adequately support their learning. Following implementation of the Joseph-RATA model, students reported consistently favorable perceptions across all evaluated domains of the structured feedback questionnaire. Although the baseline and post-intervention perceptions were assessed using different questionnaire items and were therefore not directly comparable statistically, the findings collectively suggest that learners perceived the structured feedback model to be more useful, transparent, and learner-centered than the previous feedback approach. These observations are consistent with previous studies that have highlighted the value of structured and actionable feedback in CBME [2,6].

The high mean scores observed across the structured feedback domains reflect strong learner endorsement. The educational literature consistently emphasizes that effective feedback must be specific, criterion-based, timely, and actionable [10]. The Joseph-RATA model incorporates explicit scoring descriptors linked to performance levels, thereby operationalizing principles described in evidence-based feedback guidelines that identify clarity, specificity, and alignment with observable behaviors as hallmarks of high-quality feedback [7].

Importantly, the low variability across survey responses suggests consistency of implementation across faculty members. Structured written feedback tools in objective structured clinical examination and undergraduate assessment settings have similarly demonstrated feasibility and uniformity when clear descriptors are provided [1,12]. The ability to standardize feedback delivery while maintaining individual relevance is particularly valuable in large-group teaching environments, where individualized oral feedback may be impractical.

Qualitative findings further enriched the interpretation of the quantitative results. Students emphasized improved transparency in marking, enhanced understanding of expectations, and facilitation of self-directed learning. These findings mirror the conceptual framework of feedback literacy, which identifies awareness, agency, and engagement as critical processes for effective feedback use [13]. By explicitly linking scores with performance descriptors, the Joseph-RATA model appears to enhance learner agency and reduce ambiguity.

The theme of personalization within large-group settings is particularly noteworthy. Students reported that although feedback was delivered collectively, its interpretation remained individualized and non-threatening. Evidence suggests that psychologically safe environments improve receptivity to feedback and reduce defensiveness [7]. The structured descriptor format likely minimized perceived judgment by focusing attention on performance criteria rather than on personal evaluation.

The comparatively lower ratings for the non-judgmental nature of feedback (item 7) and reduction of assessment-related anxiety (item 13) warrant consideration. Although students described the structured approach as personalized and less threatening, feedback delivered in the context of formal assessment may retain an inherently evaluative component, which could influence perceptions of judgment [14]. Similarly, the Joseph-RATA model was primarily designed to improve the clarity, transparency, and actionability of performance feedback rather than directly address examination anxiety. The relatively lower rating for anxiety reduction may therefore indicate that structured feedback alone may not fully address the broader emotional demands associated with assessment [15]. This interpretation is also consistent with the qualitative findings, in which students requested additional guidance on examination strategy, including time management, answer length, content expectations, and answer sequencing. Future iterations of the model could therefore incorporate examination-strategy guidance and, where appropriate, complementary approaches addressing assessment-related anxiety.

The strong recommendation rate parallels findings from other structured feedback interventions in undergraduate settings, in which both students and faculty expressed a preference for continued use of structured formats [1,2]. These findings suggest that structured feedback tools are both feasible and scalable within undergraduate curricula.

Within the Indian context, the National Medical Commission's CBME framework emphasizes outcome-driven learning, transparency in assessment, and structured feedback mechanisms [11]. The Joseph-RATA model aligns with these principles by clearly defining expectations, linking performance levels to competencies, and encouraging reflective improvement. By operationalizing feedback within competency domains, the model supports development of the roles of the Indian Medical Graduate, particularly those of lifelong learner and reflective practitioner.

Students also recommended the integration of time-management guidance and clearer answer-structuring expectations within the feedback tool. These suggestions reflect a shift from feedback as error correction toward feedback as performance coaching. Educational reviews have highlighted that feedback should not only correct deficiencies but also guide future task performance and self-regulated learning [13].

The present study contributes to the limited literature on structured feedback frameworks in undergraduate pharmacology within the Indian CBME context. While previous studies have explored structured and written feedback models in undergraduate education [1,2,6], fewer have examined descriptor-linked frameworks applied to theory assessments in large cohorts. The credibility of the present findings is strengthened through methodological triangulation. Three independent sources of evidence converged toward the same conclusion. First, students retrospectively reported considerable dissatisfaction with the previous feedback system. Second, quantitative evaluation following implementation demonstrated consistently high ratings across multiple domains of structured feedback. Third, qualitative findings from FGDs and faculty interviews reinforced these observations by highlighting improved transparency, clarity of expectations, self-directed learning, and fairness in assessment. The convergence of quantitative and qualitative findings enhances confidence in the educational value and acceptability of the Joseph-RATA model.

Strengths

This study possesses several strengths. It prospectively evaluated a structured feedback intervention implemented within the routine undergraduate curriculum using universal sampling of an entire student cohort. The mixed-methods design enabled triangulation of quantitative survey findings with qualitative data obtained from FGDs and faculty interviews. Simultaneous evaluation of learner and faculty perspectives, together with a 100% response rate, strengthens the credibility and educational relevance of the findings.

Limitations

The study has several limitations. It was conducted at a single institution within a single discipline, limiting generalizability. Outcomes were based primarily on participant perceptions, and objective academic performance was not evaluated. The faculty sample was small and should therefore be interpreted descriptively. As all students received the intervention, a concurrent control group was unavailable. In addition, baseline perceptions regarding previous feedback practices were assessed retrospectively and may therefore be subject to recall bias. Finally, despite voluntary participation, the possibility of social desirability bias cannot be excluded.

Implications for practice and future research

The findings of the present study support the feasibility and acceptability of implementing the Joseph-RATA structured feedback model within routine undergraduate teaching. The model aligns with the NMC's CBME framework by promoting transparency, learner engagement, reflective practice, and competency-oriented assessment. Based on the positive learner and faculty perceptions observed in this study, the Joseph-RATA modules will be progressively integrated into formative assessments across other undergraduate subjects at our institution, with discipline-specific adaptation of the rubrics and feedback descriptors. Such wider implementation has the potential to standardize feedback practices while preserving contextual relevance across disciplines.

Future research should explore the longitudinal impact of the model on examination performance, its adaptability across disciplines, and its integration with digital platforms. Given the increasing integration of electronic and digital learning resources in medical education [16], structured feedback descriptors could be embedded within institutional learning management systems to enhance accessibility and continuity. Furthermore, advances in artificial intelligence and digital educational tools may provide opportunities to automate the generation of structured feedback while preserving educational integrity [17].

## Conclusions

Effective feedback is fundamental to meaningful learning in medical education, particularly within competency-based frameworks. The present study addressed a recognized gap in structured formative feedback within undergraduate pharmacology. Implementation of the Joseph-RATA structured feedback model resulted in high learner acceptance, improved clarity of expectations, enhanced transparency in marking, and promotion of self-directed learning. Both quantitative and qualitative findings demonstrated that linking performance descriptors with structured dialogue made feedback more actionable, consistent, and learner-centered.

The model aligns with the principles of CBME by emphasizing timely, specific, and criterion-referenced feedback, thereby operationalizing best practices recommended in the educational literature. The strong recommendation rate from students and the positive faculty perceptions indicate feasibility and scalability within large undergraduate cohorts. Overall, the structured RATA approach provided a practical and sustainable framework for delivering meaningful formative feedback. Its integration into routine academic practice may strengthen reflective learning, improve engagement, and support competency development among undergraduate medical students.

## Figures and Tables

**Figure 4:**
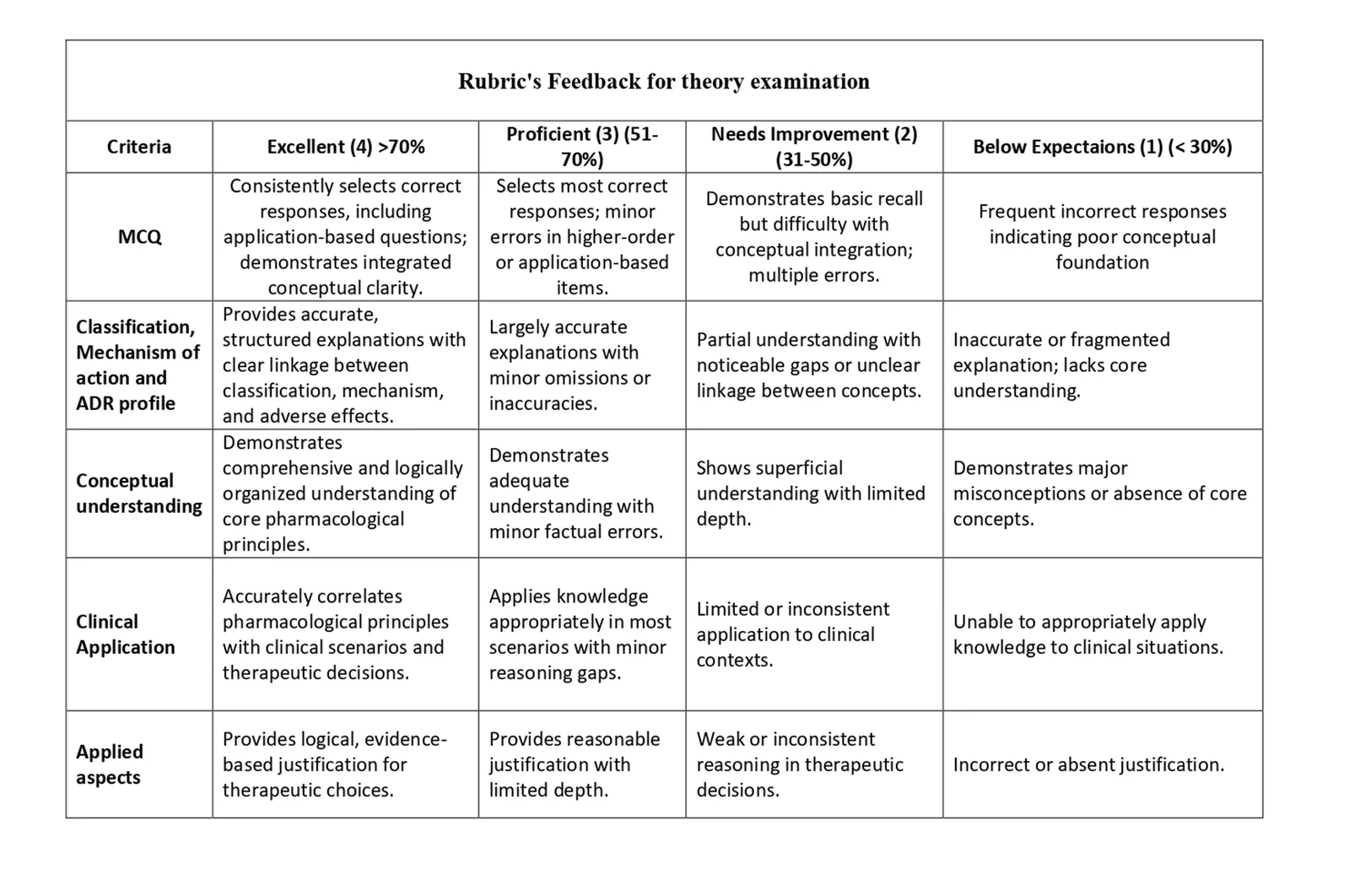
Rubric-based feedback framework for theory assessments Performance descriptors used to provide structured feedback following formative theory assessments. MCQ: Multiple-choice question; ADR: Adverse drug reaction.

**Figure 5:**
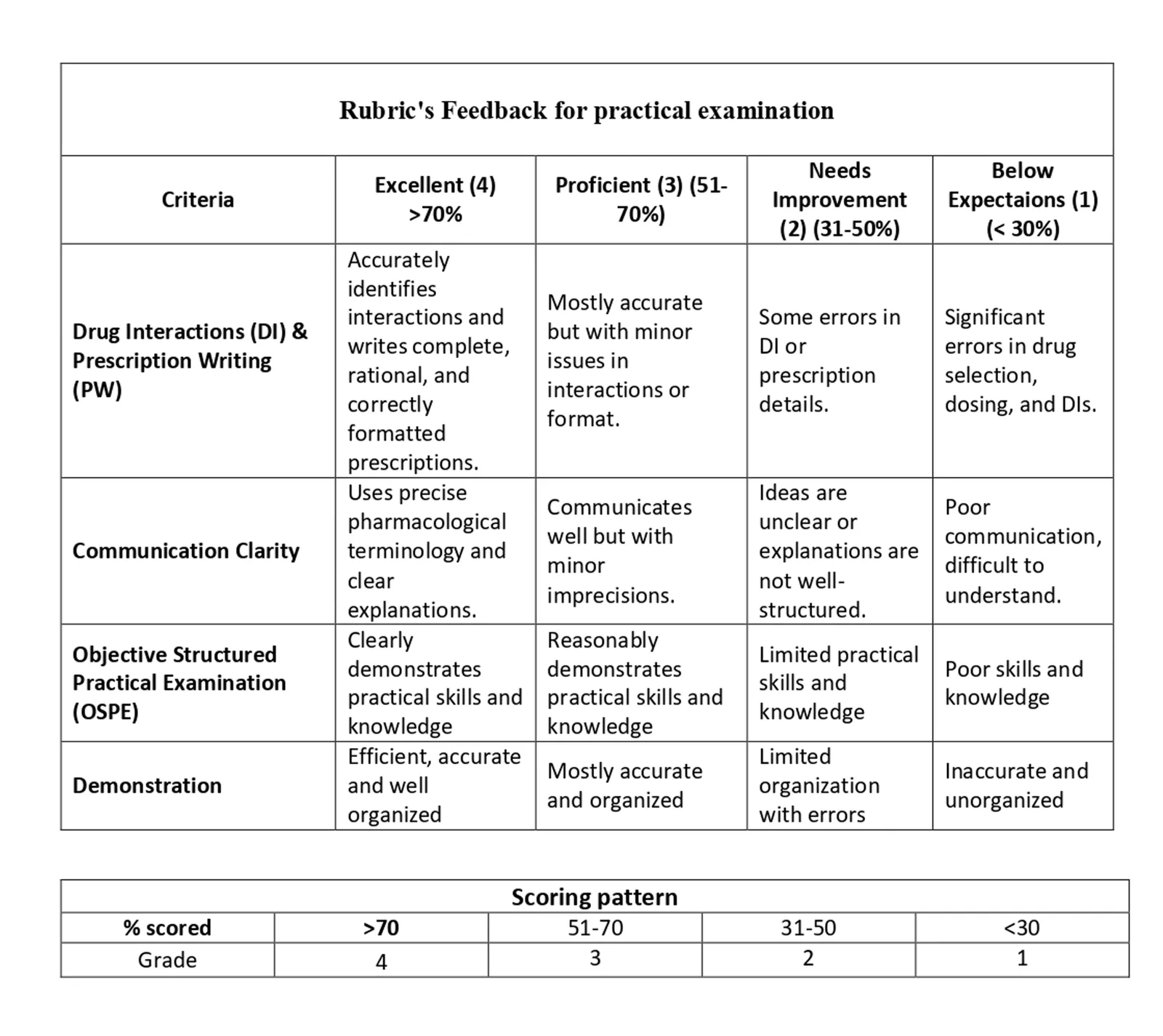
Rubric-based feedback framework for practical assessments Performance descriptors used to provide structured feedback following formative practical assessments. DI: Drug interactions; PW: Prescription writing; OSPE: Objective Structured Practical Examination.

## Appendices

Appendix 1

Appendix 2

Appendix 3

Questionnaire for Student

Your gender:

☐                    ☐                     

Male               Female            

MBBS year: Abbreviation for options (SD = Strongly Disagree, D = Disagree, N = Neutral, A = Agree, SA = Strongly Agree)

1.    The feedback provided to you “BEFORE” this method was satisfactory. ☐                    ☐                   ☐                    ☐                      ☐ 

SD                   D                    N                     A                     SA

2.    Your skill and knowledge improved “AFTER” this method was implemented. ☐                    ☐                   ☐                    ☐                      ☐ 

SD                   D                    N                     A                     SA

3.    To be a good medical graduate, a structured feedback is required to expand your medical knowledge.

☐                    ☐                   ☐                    ☐                      ☐ 

SD                   D                    N                     A                     SA

4.    To be a good clinician, a structured feedback required to improve your clinical skills

☐                    ☐                   ☐                    ☐                      ☐ 

SD                   D                    N                     A                     SA

5.    It is important to have timely feedback after assessment.

☐                    ☐                   ☐                    ☐                      ☐ 

SD                   D                    N                     A                     SA

6.    The feedback process helped me identify my strengths and areas for improvement.

☐                    ☐                   ☐                    ☐                      ☐ 

SD                   D                    N                     A                     SA

7.    The feedback sessions were non-judgmental

☐                    ☐                   ☐                    ☐                      ☐ 

SD                   D                    N                     A                     SA

8.    I am more open to receiving feedback from faculty after this method

☐                    ☐                   ☐                    ☐                      ☐ 

SD                   D                    N                     A                     SA

9.    I would prefer this method of feedback to be a regular part of future assessments.

☐                    ☐                   ☐                    ☐                      ☐ 

SD                   D                    N                     A                     SA

10.  I felt motivated to improve my performance after receiving feedback

☐                    ☐                   ☐                    ☐                      ☐ 

SD                   D                    N                     A                     SA
 

11.  The time allocated for feedback was adequate

☐                    ☐                   ☐                    ☐                      ☐ 

SD                   D                    N                     A                     SA

12.  Student satisfaction will improve with an effective feedback

☐                    ☐                   ☐                    ☐                      ☐ 

SD                   D                    N                     A                     SA

13.  This structured feedback helped reduce my anxiety related to performance and assessments

☐                    ☐                   ☐                    ☐                      ☐ 

SD                   D                    N                     A                     SA

14.  This feedback method should be repeated for future batches as part of the curriculum

☐                    ☐                   ☐                    ☐                      ☐ 

SD                   D                    N                     A                     SA  

15. Would you want this type of module to be a part of your curriculum?

·       Yes

·       No

Appendix 4

Faculty Perception Questionnaire

 (SD = Strongly Disagree, D = Disagree, N = Neutral, A = Agree, SA = Strongly Agree)

There was a need for a structured format such as the Joseph-RATA model for providing feedback to students. ☐ SD ☐ D ☐ N ☐ A ☐ SA

The faculty training adequately prepared me to implement the Joseph-RATA model. ☐ SD ☐ D ☐ N ☐ A ☐ SA

The rubric descriptors in the Joseph-RATA model were clear and easy to apply during assessment. ☐ SD ☐ D ☐ N ☐ A ☐ SA

The Ask-Tell-Ask component of the Joseph-RATA model facilitated meaningful interaction with students. ☐ SD ☐ D ☐ N ☐ A ☐ SA

The Joseph-RATA model improved transparency in marking. ☐ SD ☐ D ☐ N ☐ A ☐ SA

The Joseph-RATA model enhanced consistency in feedback across faculty members. ☐ SD ☐ D ☐ N ☐ A ☐ SA

The time required to deliver feedback using the Joseph-RATA model was manageable. ☐ SD ☐ D ☐ N ☐ A ☐ SA

The Joseph-RATA model was feasible to implement in large undergraduate batches. ☐ SD ☐ D ☐ N ☐ A ☐ SA

The Joseph-RATA model promoted reflective learning among students. ☐ SD ☐ D ☐ N ☐ A ☐ SA

Overall, I am satisfied with the Joseph-RATA feedback approach. ☐ SD ☐ D ☐ N ☐ A ☐ SA

The Joseph-RATA model can be adapted for use in other disciplines. ☐ SD ☐ D ☐ N ☐ A ☐ SA

Would you recommend continuation of the Joseph-RATA model as part of routine formative assessments? · Yes · No
